# Rational Adaptation in Using Conceptual Versus Lexical Information in Adults With Aphasia

**DOI:** 10.3389/fpsyg.2021.589930

**Published:** 2021-01-28

**Authors:** Haley C. Dresang, Tessa Warren, William D. Hula, Michael Walsh Dickey

**Affiliations:** ^1^Department of Communication Science and Disorders, University of Pittsburgh, Pittsburgh, PA, United States; ^2^Center for the Neural Basis of Cognition, Carnegie Mellon University, Pittsburgh, PA, United States; ^3^VA Pittsburgh Healthcare System, Pittsburgh, PA, United States; ^4^Department of Psychology, University of Pittsburgh, Pittsburgh, PA, United States; ^5^Learning Research and Development Center, University of Pittsburgh, Pittsburgh, PA, United States

**Keywords:** aphasia, rational adaptation, adaptation, verb naming, priming, event knowledge, co-occurrence statistics

## Abstract

The information theoretic principle of rational adaptation predicts that individuals with aphasia adapt to their language impairments by relying more heavily on comparatively unimpaired non-linguistic knowledge to communicate. This prediction was examined by assessing the extent to which adults with chronic aphasia due to left-hemisphere stroke rely more on conceptual rather than lexical information during verb retrieval, as compared to age-matched neurotypical controls. A primed verb naming task examined the degree of facilitation each participant group received from either conceptual event-related or lexical collocate cues, compared to unrelated baseline cues. The results provide evidence that adults with aphasia received amplified facilitation from conceptual cues compared to controls, whereas healthy controls received greater facilitation from lexical cues. This indicates that adaptation to alternative and relatively unimpaired information may facilitate successful word retrieval in aphasia. Implications for models of rational adaptation and clinical neurorehabilitation are discussed.

## Introduction

The language-processing system has often been viewed as relatively static and context-invariant, particularly by sentence comprehension models (e.g., Frazier, 1987; Bornkessel and Schlesewsky, 2006). However, recent evidence indicates that successful language processing, including sentence comprehension, is accomplished by an adaptive system (Ellis and Larsen-Freeman, 2009 for review; Gibson et al., 2013). There is growing evidence that the language system flexibly takes advantage of a wide array of sources of information to guide performance. These may include linguistic representations (grammatical categories, thematic roles, and lexical co-occurrence probabilities), contextual constraints, and knowledge of the relationships between words and real-world events (e.g., Caramazza and Zurif, 1976; McRae and Matsuki, 2009; Gibson et al., 2013; Kuperberg and Jaeger, 2016; Dresang et al., 2018). According to information theory, reliance on these information sources is governed by *the principle of rational adaptation* (Anderson, 1991; Howes et al., 2009), which states that a system can modify the degree to which it relies on different information sources in order to optimize behavior under different experimental conditions (e.g., Gibson et al., 2013) or disease states (Caramazza and Zurif, 1976; Gibson et al., 2015; Warren et al., 2017).

Language performance in individuals with aphasia provides a unique way to evaluate hypotheses regarding the adaptive use of information sources during language processing. People with aphasia have impairments in accessing and using linguistic information, but their stored conceptual-semantic knowledge is usually less impaired. The assumption that people with aphasia therefore rely more heavily on conceptual-semantic information undergirds both classic accounts of aphasic sentence processing (Caramazza and Zurif, 1976; Goodglass, 1976) and efficacious speech-language treatments (e.g., Boyle, 2010; Wambaugh et al., 2014; Edmonds, 2016). However, it remains unclear whether individuals with aphasia show evidence of rational adaptation during production tasks. The current study looks for evidence of rational adaptation during verb retrieval by people with aphasia. In doing so, it is one of few to investigate aphasic rational adaptation in reliance on stored representations of linguistic versus conceptual knowledge (see also Caramazza and Zurif, 1976), rather than in reliance on bottom-up linguistic input (e.g., Gibson et al., 2015; Warren et al., 2017). Verb-retrieval deficits are important to study because they are frequently observed in 70 percent of individuals with aphasia, across severity levels and syndrome classification types (Mätzig et al., 2009).

The rational adaptation principle is key to the noisy channel, or rational inference, account of sentence comprehension. According to this account, comprehenders perceive a sentence and immediately compute the probabilities associated with its possible intended messages. Their estimations of these probabilities adapt quickly to changes in the amount of noise or the reliability of cues in the context (Gibson et al., 2013). Gibson et al. (2013) demonstrated that increasing the rate of typos in an experiment led participants to rely less on linguistic form during sentence interpretation. Similarly, increasing the proportion of implausible sentences in the experiment led participants to rely less on meaning to guide sentence interpretation. Gibson et al. (2015) extended this work to adults with aphasia. They tested the hypothesis that during language comprehension, people with aphasia should rely more heavily on conceptual knowledge than healthy adults, because their linguistic impairments are more likely to introduce noise into their representations of the bottom-up linguistic input. In this study, like the 2013 one, sentence plausibility was crossed with sentence structure in such a way as to create implausible sentences that differed from plausible sentences by a small edit, and vice versa. For example, the implausible sentence *The mother gave the candle the daughter* is a single dropped *to* from the plausible sentence *The mother gave the candle to the daughter.* Greater reliance on conceptual knowledge would be shown by a stronger tendency to interpret implausible sentences like *The mother gave the candle the daughter* as if they were plausible near neighbors like *The mother gave the candle to the daughter*. This is because plausibility is conceptually driven. Gibson and colleagues showed that, like controls, people with aphasia were sensitive to the likelihood that a particular sentence structure would be distorted into its near neighbor (for example, they were more likely to stick with the literal interpretation of sentences with structures that were higher frequency or required an insertion rather than a deletion to become a plausible near neighbor). But across multiple types of sentences, people with aphasia were more likely than controls to interpret implausible sentences as their plausible near neighbors. That is, participants with aphasia showed a stronger influence of plausibility on their sentence interpretations than control participants did. This suggests they had rationally adapted to rely more heavily on conceptual knowledge, e.g., plausibility, than control participants. Warren et al. (2017) extended and replicated these findings using a different paradigm and a larger sample of people with aphasia.

These findings from experiments testing noisy channel processing in aphasia point to a flexible language processing system that is sensitive to aphasia-related changes in the reliability of cues to interpretation, including the likelihood of input distortion. But these studies have been relatively narrowly focused, in that the only language-related cue that has been tested is the form of the input, and the only outcome measure has been the ultimate interpretation of the sentence. A study by Hayes et al. (2016) tested a different kind of language-related cue, namely verb-argument requirements, during incremental comprehension. They pitted verb-argument information against plausibility in a visual-world study testing the anticipatory processing of event locations (e.g., “The child put/rode the bicycle in the park/pool.”). They found that both the argument structure requirements of verbs and the plausibility of the event location guided the anticipatory processing of neurotypical adults across the lifespan, but only plausibility influenced anticipatory processing in adults with aphasia. This is consistent with aphasia increasing reliance on conceptual plausibility knowledge. However, the small size of their sample of participants with aphasia raises concerns about power, and this evidence (like that of Gibson et al., 2015 and Warren et al., 2017) speaks only to whether rational adaptation characterizes comprehension performance in aphasia.

The current study builds on a series of studies reported in Willits et al. (2015) that investigated unimpaired language users’ reliance on language knowledge versus event knowledge across multiple tasks. The form of language knowledge they focused on is word co-occurrence frequency (Hale, 2001; Levy, 2008). We know that healthy language users utilize their stored knowledge of word co-occurrence in both comprehension and production (e.g., Wasow, 1997; Reali and Christiansen, 2007). There is also evidence that people with aphasia make use of lexical frequency and word co-occurrence information. In Gahl (2002), participants with fluent and anomic aphasia types showed sensitivity to lexical verb biases in a sentence plausibility judgment task. In a subsequent set of experiments, Dede (2013a, b) observed that the effects of lexical verb bias were greater in adults with aphasia than controls in an on-line self-paced reading task. These results suggest that word co-occurrence can influence sentence comprehension in aphasia. However, it remains unknown whether individuals with aphasia make use of word co-occurrence to facilitate naming.

Willis and colleagues (Willits et al., 2015) also tested the influence of event knowledge on language performance. In healthy adults, priming experiments have demonstrated that memory is structured such that multiple types of single-word cues allow immediate access to event knowledge (Ferretti et al., 2001; McRae et al., 2001, 2005; Hare et al., 2009). In particular, verbs prime nouns that commonly fill their event-related thematic roles (agents, patients, instruments; Ferretti et al., 2001) and vice versa (McRae et al., 2001, 2005). In addition, Hare et al. (2009) found that nouns that denote common events (e.g., trip, accident) primed objects and agents typically involved in that event (trip–luggage; accident–policeman), and that location and instrument nouns primed event-related object and agent targets. Taken together, this evidence indicates that isolated verbs, event nouns, and thematic role/participant nouns activate conceptual event knowledge, resulting in facilitated naming of related concepts. This kind of direct event-related priming has not previously been tested in people with aphasia, but Dresang et al. (2019) found an indirect relation between event knowledge and verb naming. They found that conceptual knowledge of events positively predicted performance on verb naming and argument structure production tests in a sample of people with aphasia.

These two types of knowledge, word co-occurrence and event knowledge, are not always independent given that language is used to communicate information about events in the real world. But they can be dissociated. Willits et al. (2015) conducted two corpus analyses and found that past progressive verbs co-occur more frequently with locations than do past perfect verbs. However, this varied across individual verbs. Willits et al. (2015) capitalized on this variability to create verb-location stimuli with three levels: event related pairs with high co-occurrence probability, event related pairs with low co-occurrence probability, and unrelated pairs with low co-occurrence probability. These stimuli were tested in four behavioral tasks, to investigate whether young neurotypical adults lean more heavily on different sources of information under different task conditions. In two semantic tasks, plausibility judgment (“Rate how likely it is that the event or action described typically takes place in this location.”) and semantic judgment (“Is this a location?”), results were driven by event knowledge. But in two language-production-focused tasks, primed verb naming (“Say the target word aloud.”) and sentence completion (“Mary was visiting…”), effects were driven by word co-occurrence patterns. These findings support the notion that healthy adults prioritize conceptual event versus word co-occurrence information to different degrees depending on the task demands.

The current study extends this work with the goal of investigating rational adaptation in aphasia by testing the hypothesis that: because language impairment reduces the reliability of linguistic information for people with aphasia, they will rely more heavily on event knowledge and less heavily on linguistic knowledge as compared to unimpaired adults. Given that Willits et al. (2015) found that young neurotypical participants relied heavily on word co-occurrence information in a naming task, the current study used a naming task in people with aphasia. We expected to replicate Willits and colleagues’ finding that healthy control participants exhibit stronger effects of word co-occurrence than event-relatedness on naming. But we further predicted that people with aphasia would show the opposite pattern and exhibit a larger facilitative effect of event relatedness than word co-occurrence on naming. The current study breaks new ground because evidence for rational adaptation in aphasia to date is limited to auditory sentence comprehension (Caramazza and Zurif, 1976; Schwartz et al., 1980, 1987; Gibson et al., 2015; Hayes et al., 2016; Warren et al., 2017). This study also has practical import because rational adaptation could be a mechanism behind the apparent efficacy of speech-language therapies that treat verb-retrieval deficits in people with aphasia by strengthening conceptual-semantic networks around verbs (e.g., Verb Network Strengthening Treatment [VNeST]; see Edmonds, 2016, for review). Demonstrating rational adaptation in verb naming would be a first step in showing that it may underlie these efficacious speech-language treatments and might be leveraged to develop more targeted neurorehabilitation methods, by determining what information cue types and experimental (learning) conditions facilitate verb retrieval. Finally, it contributes to studying a common, but relatively understudied, aspect of aphasia. 70 percent of individuals with aphasia experience chronic verb-retrieval deficits (Mätzig et al., 2009).

## Materials and Methods

### Participants

Participants were 17 individuals with chronic aphasia due to unilateral left hemisphere stroke and 15 age-matched neurotypical controls. All participants were (1) native English speakers, (2) able to provide informed consent, (3) 25–85 years old, (4) (premorbidly) right-handed, (5) had no significant hearing loss or vision impairment that prevented them from completing the experimental tasks, (6) had no pre-existing or subsequent brain injury/stroke (e.g., to right-hemisphere regions for individuals with aphasia), and (7) had no history of progressive neurological or psychiatric disease, drug, or alcohol dependence, or significant mood or behavioral disorder.

In addition, all neurotypical participants passed a line-bisection visual screening, a binaural pure-tone hearing screening (0.5, 1, 2, and 4 KHz at 40 dB), a Mini-Mental State Examination cognitive screen (required 27/30; Folstein et al., 1975), and Raven’s Colored Progressive Matrices non-linguistic cognitive screen (required 30/36; Raven, 1965). All individuals with aphasia were more than 6 months post-onset (range: 19–265 months; M = 95.8, SD = 62 months), had a Comprehensive Aphasia Test (CAT; Swinburn et al., 2004). Naming Modality T-score ≥ 40, and an overall mean T-score < 70. Cognitive screening and general language assessment measures, including the CAT, were already available for the participants with aphasia, who all participated in Hula et al. (2020). Participants were not recruited if their T scores were less than 30 for the CAT Cognitive Screening semantic memory or recognition memory subtests. T scores under 30 would be indicative of frank auditory, visual, motor speech, or general cognitive deficits. Demographic participant characteristics are reported in Table 1 for participants with aphasia and Table 2 for age-matched controls.

**TABLE 1:** Demographic characteristics of participants with aphasia.

**Participant ID**	**Age**	**Sex**	**Education level**	**Years of education**	**Months post-onset**	**Years post-onset**
7201	59	F	Graduate degree	20	132	11
7202	63	M	Bachelor’s degree	14	265	22.08
7203	61	F	Master’s degree	17	60	5
7204	55	M	High school	12	53	4.42
7205	52	M	High school	12	136	11.33
7206	78	F	Some graduate	13	114	9.5
7207	70	F	Some college	14	45	3.75
7208	76	M	Some college	14	138	11.5
7209	77	M	Law degree	19	53	4.42
7210	54	M	Bachelor’s degree	16	83	6.92
7211	71	M	Some college	14	26	2.17
7212	55	M	Bachelor’s degree	16	19	1.58
7213	68	M	High school	12	184	15.33
7214	53	F	Bachelor’s degree	17	81	6.75
7215	71	M	Bachelor’s degree	16	87	7.25
7216	72	M	Some college	14	60	5
7217	72	M	Some college	15	93	7.75
Summary	M = 65.12	5 F; 12 M		M = 15	M = 95.82	M = 7.99
	SD = 9.11			SD = 2.35	SD = 62	SD = 5.17

**TABLE 2:** Demographic characteristics of age-matched control participants.

**Participant ID**	**Age**	**Sex**	**Education level**	**Years of education**
7001	42	M	Tech college	14.5
7002	59	M	High school	12
7003	74	M	Bachelor’s degree	16
7004	52	M	Bachelor’s degree	16
7005	54	M	Bachelor’s degree	16
7006	57	M	High school	12
7007	72	F	Master’s degree	18
7008	64	F	Master’s degree	18
7010	74	M	Master’s degree	20
7011	68	F	Master’s degree	22
7012	72	M	Bachelor’s degree	16
7013	65	M	Law degree	19
7014	71	F	Master’s degree	17
7015	52	M	Master’s degree	22
7016	69	F	Master’s degree	18
Summary	M = 63	5 F; 10 M		M = 17.1
	SD = 9.82			SD = 3

Institutional Review Board approval was obtained, and all participants provided informed written consent and were compensated for their time.

### Materials

Experimental stimuli were adapted from existing normed stimuli for agent-, patient-, instrument-, and location-verb pairs (McRae et al., 2005). We developed items that paired 48 target verbs from McRae et al. (2005) with each of three kinds of noun primes. In the event-related condition, the primes were nouns that were strongly associated with the target verb’s event but rarely appeared within four words of the verb in COCA’s Wikipedia corpus (*pencil–WRITE*). Event-related primes were drawn from McRae et al. (2005) or from the USF Free Association Norms (Nelson et al., 1998) and consisted of agents, patients, instruments, or locations strongly associated with the target verb’s event. Only seven event-related primes were among the top 100 noun collocates for their target verb (Maximum = 50th, M = 65th). In the lexical co-occurrence condition, the primes were nouns that co-occurred frequently with the target verbs but were not strongly associated with the target verb’s event (*name–WRITE*). Lexical co-occurrence primes were selected from the nouns that most frequently appear within four words of the target verb in COCA’s Wikipedia corpus (Davies, 2008). We chose the highest-ranked (M = 7–8th, range: 1st–25th) collocate that: (1) was not a paradigmatic participant in the verb’s event (i.e., did not appear in McRae et al., 2005 or Nelson et al., 1998 norms), (2) did not form a compound with the verb (e.g., *board-WALK; school-WORK*), and (3) was not a high collocate of many verbs. Two of the authors confirmed these via independent judgments. In the baseline control condition, the primes were nouns that were neither associated with the verb’s event nor often appeared near the verb (*water–WRITE*). They were generated by reassigning event-related primes to targets such that semantic relationships were minimized. Semantic distance between cue and target words was calculated using snaut semantic distance measure (Landauer and Dumais, 1997; Mandera et al., 2017) to confirm that lexical co-occurrence and baseline conditions were matched for lexical-semantic relatedness between cue and target words (*t*-statistic = −0.41; *p*-value = 0.68). Prime noun word length was balanced across conditions (all p’s > 0.26). Following a Latin square design, conditions were counterbalanced and pseudorandomized across three presentation lists. See Supplementary Materials for a complete stimulus list that includes individual item properties.

### Testing Procedures

Each participant completed all three presentation lists, interleaved with other behavioral experiments with different tasks. Every presentation list began with six practice trials, followed by 48 experimental trials. Each trial began with a central fixation cross displayed for 25 milliseconds, followed by a noun prime (in lower-case blue letters) for 450 milliseconds, followed by a central mask (&&&&&&&) for 50 milliseconds, and then the verb naming target (in upper-case black letters) remained on the screen until the participant provided a response or indicated inability to do so. An audio click was presented simultaneously with the target verb for the purpose of manual measurement of naming latencies. Because naming is challenging for people with aphasia and they do not always process incoming linguistic information efficiently (Goodglass and Wingfield, 1997; Faroqi-Shah et al., 2010; Silkes et al., 2020), we used a relatively long prime duration (longer than the standard 200 milliseconds for lexical decision tasks). In addition, within each presentation list, we blocked items according to whether the primes most naturally preceded the verb (i.e., event prime agents and instruments; preceding collocates; e.g., *actor–PERFORM, ax–CHOP*) or followed it (i.e., event prime patients and locations; following collocates; e.g., *customer–SERVE, gym–EXERCISE*). Following McRae et al. (2005), trials were separated by a 1,500-millisecond blank screen. Participants were instructed to name the target verb aloud as quickly and accurately as possible. An external microphone recorded naming responses in Audacity^®^, and accuracy and latency measurements were coded by hand.

Accuracy and response time were the dependent variables. Trained raters followed procedures outlined by the Philadelphia Naming Test (Roach et al., 1996) in order to determine the first complete attempt, which was then scored for both accuracy and latency. Accuracy was coded as correct or incorrect. Participants with aphasia who had concomitant motor speech impairments (e.g., dysarthria, speech apraxia) were allowed one sound omission, addition, or substitution per response when considering correctness (Roach et al., 1996). Response time (latency) was measured in milliseconds from the time in which the target word was displayed (with audio click) until the participant began to produce their first complete response. These scoring procedures followed the conventional procedures used for the Philadelphia Naming Test (Roach et al., 1996). Two raters measured the critical time points and calculated the naming latency for each trial. They had 93.77 percent agreement on a randomly selected sample of 10 percent of the items (ratings within 50 milliseconds of each other constituted agreement). The raters discussed these discrepancies and reached 100 percent agreement. The degree of priming was measured by comparing the latency of event and lexically related word pairs to baseline, unrelated trials.

### Analyses

Data were analyzed using Bayesian mixed effects regression models, which were created in the Stan computational framework (Carpenter et al., 2017; http://mc-stan.org/) accessed with the brms package (Bürkner, 2017). Trial-level naming accuracy served as the outcome variable for two logit-link bernoulli family models, and trial-level naming response time served as the outcome variable for two ex-gaussian family models. Model 1 examined naming accuracy between participant groups; Model 2 examined naming response time between groups; Model 3 examined accuracy in participants with aphasia; and Model 4 examined response time in participants with aphasia. Estimates of facilitation under each prime condition (baseline, event-related, and lexical co-occurrence) were assessed in terms of the assumptions of normality, homoscedasticity, linearity, and the presence of outliers. To address outliers and to achieve model convergence, latency observations above the 95th percentile for each group were trimmed. From 3,200 trials, 89 trials were trimmed (2.8% of the original data), resulting in a total of 3,111 observations across both groups. Finally, only accurate trials were examined in Model 2 and Model 4, for which response time was the dependent variable (Forster, 1976).

The model structures are discussed below. Each parameter was given dispersed starting values and a vague prior, thus allowing the Bayesian estimation process to explore the full parameter space and provide conservative estimates of posterior distributions (McElreath, 2020). For each model, four Hamiltonian Markov chain Monte Carlo (MCMC) chains were run for 20,000 samples, with half of the iterations discarded as warm-up and 10,000 iterations monitored for convergence and parameter estimation. There was no thinning and no divergent transitions for any of the models. For each model, MCMC convergence was assessed graphically by inspection of the autocorrelation and trace plots, as well as statistically using the Gelman-Rubin potential scale reduction statistic (R̂) and the number of effective samples. The R̂ statistic is a ratio of the variance within each chain to the variance pooled across chains. *R̂* values close to 1 indicate satisfactory convergence of the chains to a stable distribution (Gelman et al., 2013). ESS factors out the autocorrelation in the observed MCMC chains and estimates the number of independent samples that would achieve the same degree of precision for the parameter estimates (Carpenter et al., 2017). Large ESS values indicate satisfactory convergence. The posterior distributions are summarized by the estimated parameters and 95% highest density credible intervals (HDI). The HDI is comparable to the frequentist confidence interval and is determined as the narrowest interval containing the assigned proportion of the posterior distribution’s probability mass within which all values have a higher probability density than any values outside the interval (see Fergadiotis et al., 2019 for further explanation).

*Post hoc* pairwise comparisons were conducted using the emmeans package in R (Lenth, 2020) for Models 1–2 in order to evaluate the reliability of every potential condition-specific priming effect for both groups of participants.

First, naming accuracy was compared between participant groups with and without aphasia. The outcome variable was trial-level verb naming accuracy. Fixed effects were group assignment (participants with aphasia coded as 0 versus neurotypical controls coded as 1) and prime condition (event-relatedness versus baseline; and lexical co-occurrence versus baseline), and two interaction effects (group *x* event condition; group *x* lexical condition). The effect of prime condition was dummy coded with the baseline condition as the reference level. Specifically, the prime condition fixed effect was coded with two contrasts across the three levels of the variable, such that each condition of interest was compared to the baseline prime condition. Random intercepts were included for subjects and items. Random slopes were included for condition within subjects and group within items. More complex random effects structures failed to converge.

Second, naming response time was compared between participant groups with and without aphasia. Fixed effects and random effects structures were the same as for Model 1, but the outcome variable was response time (latency) from word presentation to participant response, in milliseconds.

Third, naming accuracy was examined in greater detail for participants with aphasia. The outcome variable was trial-level verb naming accuracy. Each prime condition was a fixed effect. Prime conditions were coded the same way as for Models 1 and 2. Random intercepts were included for subjects and items. Random slopes were included for condition within subjects and aphasia severity within items.

Fourth, naming response time was examined in greater detail for participants with aphasia. Fixed effects and random effects structures were the same as for Model 3, but the outcome variable was response time (latency) from word presentation to participant response, in milliseconds.

## Results

Descriptive statistics of group level accuracy and response time across each prime condition are reported in Table 3. The trace plots for all parameters demonstrated rapid convergence and were stationary relative to the parameter means. The autocorrelation plots corroborated this assessment and showed minimal autocorrelation for all four models. These plots and all posterior predictive checks are provided in the Supplementary Material. The R̂ statistic and number of effective samples for each parameter indicated satisfactory convergence and MCMC mixing. These statistics are reported in Tables 4–7. Tables 4–7 also provide the point estimates and 95% credible intervals for each parameter. The posterior predictive checks and histograms of the posterior distributions for the estimates of interest are provided below. Only differences where less than 20% of the posterior probability distributions did not overlap zero are interpreted below (Hair et al., 2009; Hazelrigg, 2009).

**TABLE 3:** Descriptive statistics of group level accuracy (percent correct) and response time (seconds) across prime conditions.

**Prime Condition**		**Participants with aphasia**		**Control participants**		**Grand total**	
		**M**	**SD**	**M**	**SD**	**M**	**SD**
Baseline	Accuracy	0.774	0.419	0.996	0.064	0.885	0.242
	Latency	0.779	0.271	0.598	0.139	0.689	0.205
Event	Accuracy	0.805	0.397	0.990	0.098	0.898	0.248
	Latency	0.822	0.271	0.596	0.159	0.709	0.215
Lexical	Accuracy	0.792	0.406	0.999	0.037	0.896	0.222
	Latency	0.822	0.280	0.589	0.127	0.706	0.204
Grand total	Accuracy	0.790	0.407	0.995	0.067	0.893	0.237
	Latency	0.814	0.269	0.593	0.132	0.701	0.208

*These values reflect the descriptive statistics after excluding outliers.*

**TABLE 4:** Model 1 primed naming accuracy population-level effects for participants with aphasia and age-matched control participants.

	**Estimate**	**Est. error**	**Lower 95% HDI**	**Upper 95% HDI**	**R̂**	**Bulk ESS**	**Tail ESS**
(Intercept)	1.84	0.54	0.79	2.94	1	1836	3811
Group	5.41	1.22	3.06	7.83	1	3055	4291
Event-related prime	0.3	0.21	−0.14	0.69	1	8274	6879
Lexical co-occurrence prime	0.26	0.26	−0.22	0.80	1	6277	5352
Group: Event prime	−1.32	0.88	−3.13	0.37	1	6724	5599
Group: Lexical prime	1.35	1.51	−1.32	4.46	1	8408	5913

*HDI, Highest density credible interval. R̂ = The potential scale reduction factor on split chains (at convergence, R̂ = 1). ESS, Effective sample size.*

**TABLE 5:** Model 1 naming accuracy pairwise comparisons.

**Contrast**	**Estimate**	**Lower 95% HDI**	**Upper 95% HDI**
Aphasia baseline – control baseline	–5.337	–7.831	–3.060
Aphasia baseline – aphasia event	–0.293	–0.686	0.144
Aphasia baseline – control event	–4.327	–6.557	–2.349
Aphasia baseline – aphasia lexical	–0.251	–0.799	0.217
Aphasia baseline – control lexical	–6.851	–10.345	–3.896
Control baseline – aphasia event	5.040	2.844	7.576
Control baseline – control event	0.979	–0.623	2.794
Control baseline – aphasia lexical	5.074	2.791	7.621
Control baseline – control lexical	–1.485	–4.736	1.068
Aphasia event – control event	–4.029	–6.3	–2.140
*Aphasia event – aphasia lexical*	*0.041*	−*0.519*	*0.549*
Aphasia event – control lexical	–6.545	–10.089	–3.635
Control event – aphasia lexical	4.075	2.046	6.284
*Control event – control lexical*	−*2.453*	−*5.5*	*0.040*
Aphasia lexical – control lexical	–6.598	–10.092	–3.616

*HDI = Highest density credible interval. Median point estimates displayed. Results are given on the log odds ratio scale.*

**TABLE 6:** Model 2 primed naming response time population-level effects for participants with aphasia and age-matched control participants.

	**Estimate**	**Est. error**	**Lower 95% HDI**	**Upper 95% HDI**	**R̂**	**Bulk ESS**	**Tail ESS**
(Intercept)	−0.272	0.052	−0.045	−0.009	1	1928	3531
Group	−0.274	0.072	−0.415	−0.133	1	1795	3438
Event-related prime	0.008	0.018	−0.027	0.0431	1	4452	6071
Lexical co-occurrence prime	0.000	0.018	−0.034	0.036	1	4360	6273
Group : Event prime	−0.011	0.015	−0.041	0.016	1	12723	8405
Group : Lexical prime	−0.009	0.014	−0.036	0.019	1	12647	7708

*HDI, Highest density credible interval. R̂ = The potential scale reduction factor on split chains (at convergence, R̂ = 1). ESS, Effective sample size.*

**TABLE 7:** Model 2 naming response time pairwise comparisons.

**Contrast**	**Estimate**	**Lower**	**Upper**
		**95% HDI**	**95% HDI**
Aphasia baseline – control baseline	0.0484	0.0237	0.0746
Aphasia baseline – aphasia event	−0.0026	−0.0073	0.0019
Aphasia baseline – control event	0.0488	0.0238	0.0754
Aphasia baseline – aphasia lexical	−0.0008	−0.0054	0.0038
Aphasia baseline – control lexical	0.0501	0.0244	0.0757
Control baseline – aphasia event	−0.0510	−0.0762	−0.0250
Control baseline – control event	0.0002	−0.0046	0.0049
Control baseline – aphasia lexical	−0.0492	−0.0758	−0.0248
Control baseline – control lexical	0.0015	−0.0035	0.0062
Aphasia event – control event	0.0513	0.0243	0.0753
*Aphasia event – aphasia lexical*	*0.0017*	−*0.0029*	*0.0065*
Aphasia event – control lexical	0.0525	0.0264	0.0778
Control event – aphasia lexical	−0.0495	−0.0759	−0.0244
*Control event – control lexical*	*0.0013*	−*0.0038*	*0.0061*
Aphasia lexical – control lexical	0.0508	0.0247	0.0758

*HDI, Highest density credible interval. Median point estimates displayed.*

### Model 1: Primed Naming Accuracy Between Participant Groups

**FIGURE 1:**
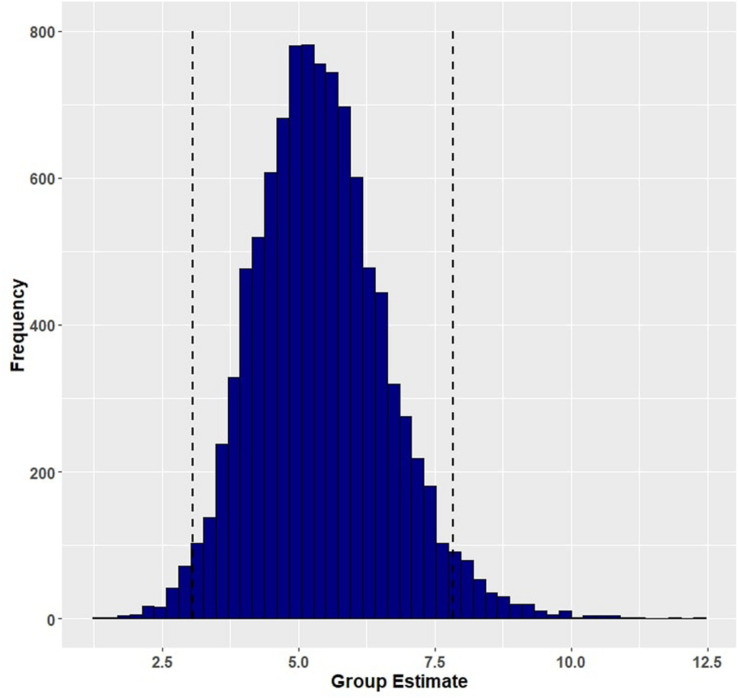
Posterior distribution and 95% highest density intervals (HDIs) of the fixed effect of group from Model 1 (primed accuracy for participants with aphasia and healthy controls). Dashed lines mark the 95% highest density intervals (HDIs) for the posterior distribution.

**FIGURE 2:**
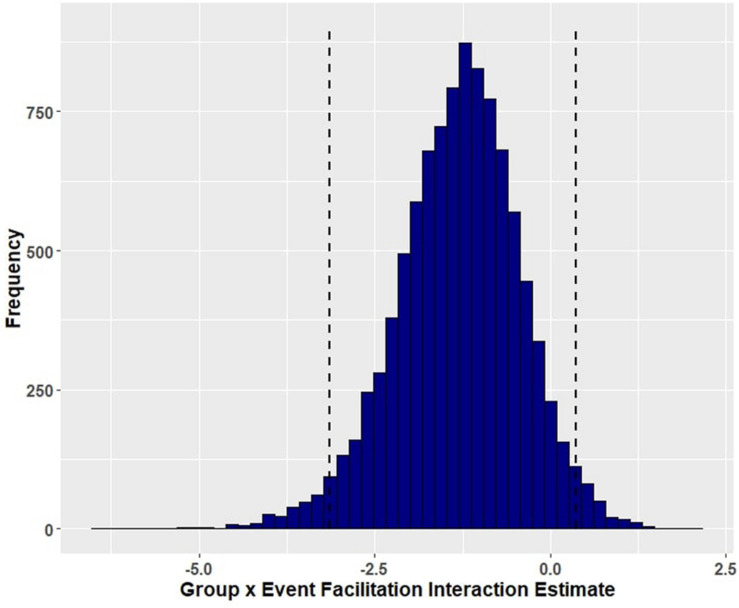
Posterior distribution and 95% highest density intervals of the interaction effect of group and event-related facilitation from Model 1 (primed accuracy for participants with aphasia and healthy controls). Dashed lines mark the 95% highest density intervals (HDIs) for the posterior distribution.

**FIGURE 3:**
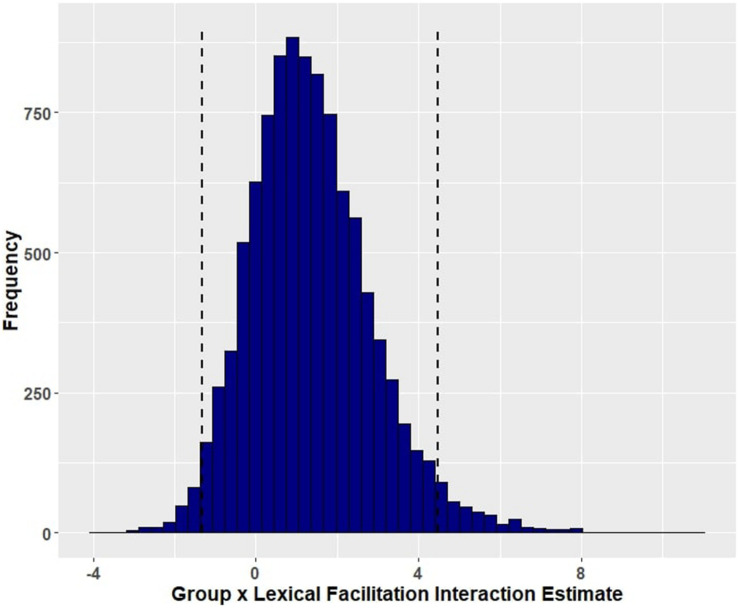
Posterior distribution and 95% highest density intervals of the interaction effect of group and lexical co-occurrence facilitation from Model 1 (accuracy for participants with aphasia and healthy controls). Dashed lines mark the 95% highest density intervals (HDIs) for the posterior distribution.

Group (aphasia versus control) reliably predicted trial-level primed naming accuracy (β = 5.41, EE = 1.22, and 95% HDI = [3.06, 7.83]), with participants with aphasia (M = 0.790, SD = 0.407) performing less well than controls (M = 0.995, SD = 0.067). Figure 1 shows the posterior probability distribution for the group effect. Furthermore, group interacted with prime condition in predicting naming accuracy, such that aphasia amplified the facilitation of event-related cues (β = −1.32, EE = 0.88, and 95% HDI = [−3.13, 0.37]) but lack of aphasia (i.e., the control group) amplified the effect of lexical co-occurrence cues (β = 1.35, EE = 1.51, and 95% HDI = [−1.32, 4.46]). Although both of these credible intervals overlap with zero, there is a 94.57 percent chance that the interaction between group and event facilitation is less than zero (Figure 2), and an 82.62 percent chance that the group and lexical co-occurrence interaction is greater than zero (Figure 3). This suggests that the observed interaction between group and event facilitation was robust, but the interaction between group and lexical co-occurrence facilitation was relatively unreliable. Based on *post hoc* pairwise comparisons, neurotypical controls received greater priming following lexical co-occurrence cues than event-related cues (β = −2.45, 95% HDI = [−5.5, 0.04]). This comparison did not show robust differences in participants with aphasia. The full set of results is reported in Table 4. The full set of pairwise comparisons is reported in Table 5.

### Model 2: Primed Naming Response Time Between Participant Groups

**FIGURE 4:**
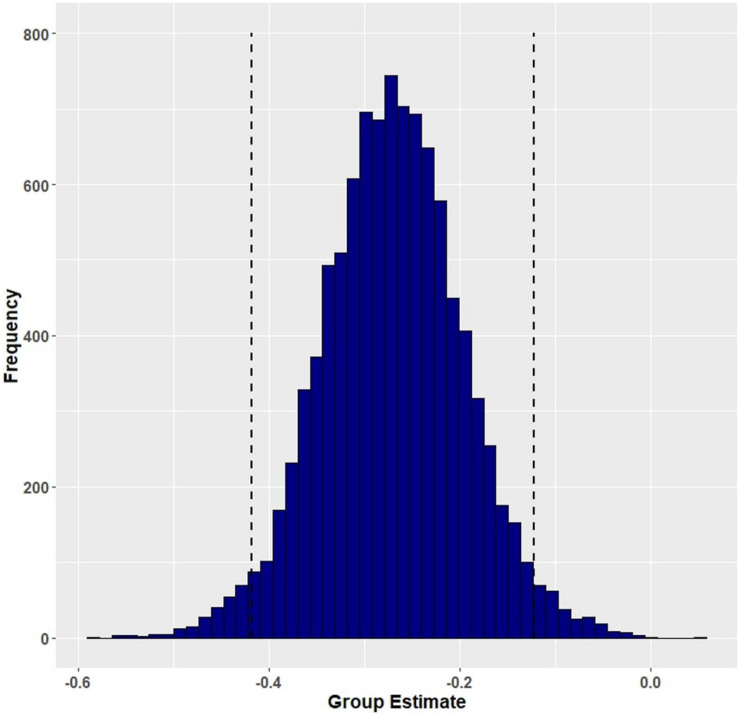
Posterior distribution and 95% highest density intervals (HDIs) of the fixed effect of group from Model 2 (primed response time for participants with aphasia and healthy controls). Dashed lines mark the 95% highest density intervals (HDIs) for the posterior distribution.

Group (aphasia versus control) reliably predicted trial-level primed naming response time (β = −0.274, EE = 0.072, and 95% HDI = [−0.415, −0.133]; Figure 4), with participants with aphasia (M = 0.814 s, SD = 0.269) performing slower than controls (M = 0.593 s, SD = 0.132). The main effects of the prime conditions and their interactions with group were small and not credibly different from zero. Based on *post hoc* pairwise comparisons, neither neurotypical controls nor participants with aphasia showed robust differences in response time following lexical co-occurrence versus event-related cues. The full set of results is reported in Table 6. The full set of pairwise comparisons is reported in Table 7.

### Model 3: Primed Naming Accuracy in Participants With Aphasia

**FIGURE 5:**
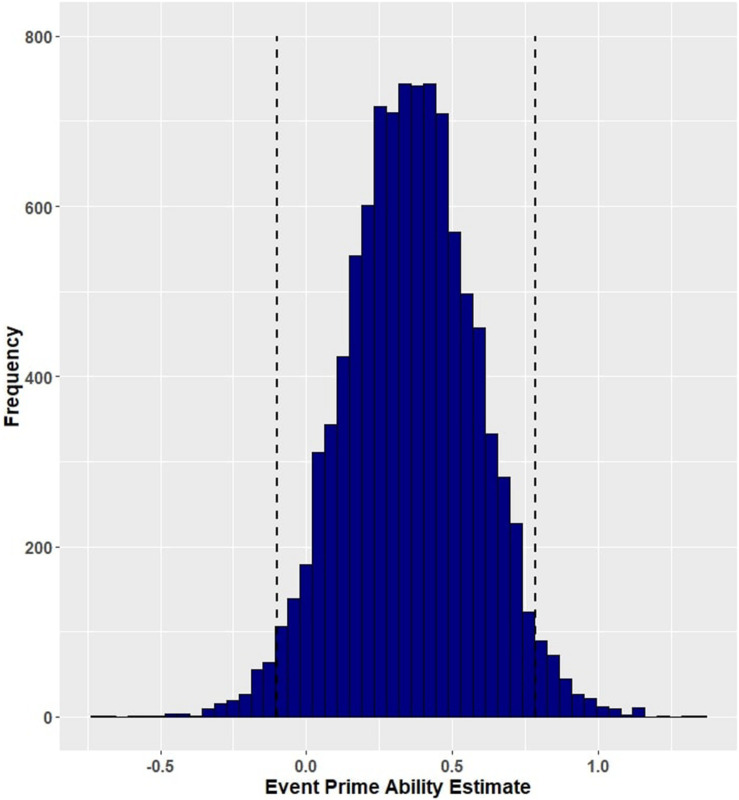
Posterior distribution and 95% highest density intervals (HDIs) of the fixed effect of event-related facilitation from Model 3 (primed accuracy for participants with aphasia). Dashed lines mark the 95% highest density intervals (HDIs) for the posterior distribution.

**FIGURE 6:**
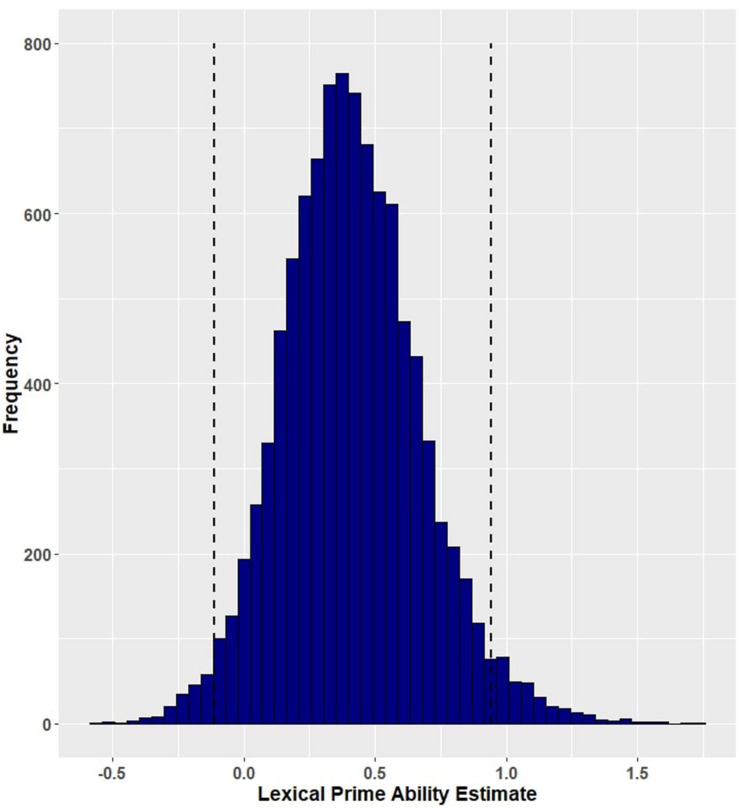
Posterior distribution and 95% highest density intervals (HDIs) of the fixed effect of lexical co-occurrence facilitation from Model 3 (primed accuracy for participants with aphasia). Dashed lines mark the 95% highest density intervals (HDIs) for the posterior distribution.

Both prime conditions predicted naming accuracy in participants with aphasia, with individuals producing more correct responses after both event-related (M = 0.805, SD = 0.397) and lexical co-occurrence primes (M = 0.792, SD = 0.406), as compared to unrelated baseline (M = 0.774, SD = 0.419). Although the 95% credible intervals for both of these effects overlap with zero, 94.69 percent of the posterior probability distribution for event primes (β = 0.36, EE = 0.23, and 95% HDI = [−0.10, 0.78], Figure 5) and 95.02 percent of the posterior distribution for lexical primes (β = 0.41, EE = 0.27, and 95% HDI = [−0.11, 0.94], Figure 6) exceed zero. The full set of results is reported in Table 8.

**TABLE 8:** Model 3 primed naming accuracy population-level effects for participants with aphasia.

	**Estimate**	**Est. error**	**Lower 95% HDI**	**Upper 95% HDI**	**R̂**	**Bulk ESS**	**Tail ESS**
(Intercept)	1.66	0.60	0.47	2.87	1	1347	2349
Event-related prime	0.36	0.23	−0.10	0.78	1	8469	6814
Lexical co-occurrence prime	0.41	0.27	−0.11	0.94	1	5683	5312

*HDI, Highest density credible interval. R̂ = The potential scale reduction factor on split chains (at convergence, R̂ = 1). ESS, Effective sample size.*

### Model 4: Primed Naming Response Time in Participants With Aphasia

No reliable priming in response time was observed in participants with aphasia for either event-related (β = 0.017, EE = 0.020, and 95% HDI = [−0.024, 0.056]) or lexical co-occurrence conditions (β = 0.010, EE = 0.017, and 95% HDI = [−0.024, 0.044]). The full set of results is reported in Table 9.

**TABLE 9:** Model 4 primed naming response time population-level effects for participants with aphasia.

	**Estimate**	**Est. error**	**Lower 95% HDI**	**Upper 95% HDI**	**R̂**	**Bulk ESS**	**Tail ESS**
(Intercept)	−0.155	0.083	−0.048	0.022	1	1613	3030
Event-related prime	0.017	0.020	−0.024	0.056	1	9456	6504
Lexical co-occurrence prime	0.010	0.017	−0.024	0.044	1	10178	7234

*HDI, Highest density credible interval. R̂ = The potential scale reduction factor on split chains (at convergence, R̂ = 1). ESS, Effective sample size.*

## Discussion

The purpose of this study was threefold. First, it aimed to replicate and extend findings from Willits et al. (2015) that indicate naming is a language-focused task in which healthy language users prioritize knowledge of word co-occurrence over conceptual event relatedness. Second, it examined the hypothesis, grounded in rational adaptation, that during verb naming adults with aphasia would rely more heavily on conceptual event-related cues and less heavily on lexical co-occurrence cues, compared to neurotypical controls. Third, aphasic behavior was examined more closely to assess differences in conceptual versus lexical facilitation within the sample of individuals with aphasia. The findings are summarized below, and their implications are discussed in relation to rational adaptation hypotheses and potential clinical directions moving forward.

First, our results from neurotypical controls were broadly consistent with findings from Willits et al. (2015), who observed that participants showed robust facilitation from frequently co-occurring words in naming tasks. The current sample of older neurotypical adults showed similar patterns to Willits and colleagues’ college-aged participants, with greater facilitation of naming in lexical-prime conditions compared to event-related conditions. This is confirmed by the pairwise comparison results. However, in the current study, these patterns appeared in accuracy rather than latency measures. Our speculation is that this might be driven by a speed-accuracy trade off, given both the high variability in latency in the current sample and previous evidence that older adults are likely to prioritize accuracy over speed (Ratcliff et al., 2004; Starns and Ratcliff, 2010). These findings suggest that unimpaired language users prioritize linguistic information (specifically, word co-occurrence frequency information) more than conceptual cues when performing naming tasks. This is consistent with findings from language production studies showing that wordform retrieval is especially sensitive to lexical frequency effects (e.g., Jescheniak and Levelt, 1994) and that high-frequency word collocations speed processing (McDonald and Shillcock, 2003; Arnon and Snider, 2010; Smith and Levy, 2013). Our results are also consistent with evidence supporting task-based rational adaptation, which contends that language users rely on the most informative source of knowledge to optimize their behavior on the task at hand (Anderson, 1991; Howes et al., 2009).

Next, we examined the effect of aphasia on primed verb naming. As expected, adults with aphasia consistently named verbs more slowly and less accurately than controls for all prime conditions. This is consistent with a large body of literature that demonstrates verb-retrieval deficits in individuals with aphasia (e.g., Berndt et al., 1997; Jonkers and Bastiaanse, 2007; Rofes et al., 2015). Response latencies showed no other effects, but verb retrieval accuracy did. Importantly, presence of aphasia interacted with prime condition in predicting verb retrieval accuracy. Participants with aphasia received an amplified facilitation effect, or greater priming, from conceptual event-related cues compared to the control group. This group by conceptual priming interaction effect was strongly reliable, with approximately 95% of the posterior probability distribution >0. There was a weaker effect in the opposite direction for lexical co-occurrence (83% of the posterior probability distribution >0): the control group received somewhat greater priming from lexical co-occurrence cues compared to participants with aphasia. However, models that examined performance only in participants with aphasia found robust facilitation effects of both conceptual event and lexical co-occurrence cues. These accuracy results extend evidence from healthy adults to individuals with aphasia: nouns prime verbs that denote events in which the nouns are commonly involved (e.g., McRae et al., 2005, 2001). This extension is critical because it highlights the importance of conceptual event knowledge in disordered language processing, which is consistent with the hypothesized mechanisms underlying efficacious speech-language treatments targeting verbs (e.g., VNeST: Edmonds, 2016; see further discussion below). Of note, the relatively unreliable interaction suggesting that lexical co-occurrence priming might be stronger in the control group than in participants with aphasia is not consistent with previous evidence suggesting that aphasia may magnify the effects of lexical frequency on language performance (Gahl, 2002; Dede, 2013a,b).

Taken together, the findings of this experiment are consistent with previous evidence of rational adaptation in aphasia and suggest that the evidence base may extend beyond sentence comprehension to verb naming. In contrast to previous investigations of rational adaptation in aphasia, this study examined stored knowledge of linguistic representations – specifically, stored knowledge of word co-occurrences – rather than bottom-up linguistic input, such as the literal sentence form (Gibson et al., 2015; Warren et al., 2017). Another critical contribution of this study is that it separately examines automatic facilitatory effects of linguistic and conceptual information types, which are independent of one another in this study design. Much of the previous evidence that is consistent with rational adaption in aphasia could be explained by the fact that people with aphasia show less reliance on linguistic knowledge than neurotypicals (e.g., Hayes et al., 2016; Warren et al., 2017). This prediction is not unique to rational adaptation, nor is it surprising given that aphasia, by definition, impairs language. For example, although linguistic and conceptual knowledge were also independent in the study by Hayes et al. (2016), they only found evidence that people with aphasia relied less on linguistic knowledge than neurotypical controls did. The current study goes beyond this in showing an increase in the use of conceptual knowledge for people with aphasia. Although overall naming performance was poorer in people with aphasia, they showed greater priming from conceptually related words than neurotypical controls did. To be clear, this finding does not necessitate rational adaptation; it could be the case that impairing one type of knowledge could change the relative utility of other types of knowledge for a structural reason, for example because one source of knowledge had been inhibiting another. Still, rational adaptation provides a straightforward and elegant account of these data.

If rational adaptation is driving these effects, assessing the mechanisms that underlie it and the potential tradeoffs between conceptual and lexical information will be informative as to what cognitive processes or routes rational adaptation might be operating over. For example, it could be reweighting different routes to lexical access, or alternatively, successive stages of lexical access. If it is reweighting lexical-access routes, the current findings may be evidence that the conceptual system – which some grounded-cognition-inspired models of meaning (Kelter and Kaup, 2012) and highly interactive/interconnected connectionist models of lexical representation (Plaut et al., 1996) have argued provides an indirect, alternate, and typically less efficient route to access lexical wordform information – is a relatively more efficient route to wordform access for people with aphasia. If rational adaptation is re-weighting inputs to successive stages of lexical access, then the nature of a lexical-access deficit may affect how successful rational adaptation is. Individuals with aphasia can experience deficits to different stages of lexical access, affecting either conceptual-to-lexical or lexical-to-phonological mapping, or both (Foygel and Dell, 2000). Individuals with more impaired conceptual-to-lexical mapping (s-weight) might receive less priming from conceptual event-related cues than individuals with relatively spared lexical-semantic processing. Of note, the degree of lexical-semantic or lexical-phonological impairment is associated with neurological variability such as lesion site and white-matter connectivity (Dell et al., 2013; Hula et al., 2020); this neurological variability may underlie person-level variation in degree of conceptual priming. Further research is needed to assess potential mechanisms that underlie the role of conceptual information in aphasic language processing.

In addition, rational adaptation predicts that increased damage to the language system would result in increased adaptive reliance on conceptual information types in people with aphasia. Applying this prediction to the current study, we would expect that aphasia severity would interact with information cue type, such that greater severity would amplify the facilitation effects of conceptual event-related cues but reduce the effects of lexical cue facilitation on verb naming. In the current investigation, overall aphasia severity was not included as a covariate predictor due to its multicollinearity with fixed effects of greater theoretical interest, such as the degree of facilitation from different information cue types. Because including aphasia severity in our models attenuated the magnitude of facilitation effects, our analyses were unable to test this prediction in the current (limited) sample. This potential limitation and the relatively small magnitude effects highlight the need for larger samples of participants with aphasia in future studies.

It is also the case, as suggested in Silkes et al. (2020), that the level of linguistic task complexity could also contribute to whether and to what degree an individual with aphasia might rely on conceptual information. Silkes et al. (2020) hypothesized that more complex tasks may be associated with decreased efficiency in engaging linguistic representations, prompting greater recruitment of more broadly distributed representations such as conceptual ones. Future work might therefore examine linguistic tasks that vary in complexity, for example comparing potential adaptation during (speeded) primed verb naming to untimed sentence completion tasks (Willits et al., 2015). The mechanisms underlying rational adaptation may be informed by a more thorough characterization of the locus and severity of behavioral and neurological impairments in individuals who receive facilitation from conceptual information during lexical access. In addition, future research might examine whether adults with aphasia show evidence of rational adaptation during language production with higher ecological validity, such as connected discourse.

Finally, the current findings may provide new evidence for mechanisms involved in efficacious aphasia interventions. A key finding from this study is that participants with aphasia exhibited a greater degree of naming facilitation from conceptual cues than neurotypical controls did. This result has critical implications for aphasia rehabilitation, because it aligns with the hypothesized mechanism of action for speech-language treatments like Semantic Feature Analysis (SFA; Boyle, 2010), SFA for Actions (Wambaugh et al., 2014), and Verb Network Strengthening Treatment (VNeST; Edmonds, 2016). Specifically, these treatments systematically activate information conceptually related to target words, based on evidence for bidirectional facilitation effects between event-related verbs and thematic roles (Ferretti et al., 2001; McRae et al., 2005). These interventions promote improved lexical retrieval ability for treated nouns (SFA) and verbs (SFA for Actions, VNeST), and there is evidence that improvements can generalize beyond trained items to the lexical retrieval of untreated words, sentences, and performance in connected discourse (e.g., Rider et al., 2008; Edmonds, 2016; Quique et al., 2019). Our rational adaption findings thus demonstrate the likely mechanism driving conceptual/semantic-based aphasia rehabilitation: If people with aphasia already exhibit reliance on conceptual information to retrieve words, then treatment can take advantage of this established mechanism by strengthening conceptually driven activation/retrieval processes. Future efforts to characterize the specific psycholinguistic and neurocognitive systems involved in this adaptation and to identify the types of patients who are most likely to engage adaptive strategies to rely more on conceptual knowledge will advance both our theoretical and clinical approaches to aphasia rehabilitation.

## Conclusion

This study found evidence suggesting that individuals with aphasia may rationally adapt to their language impairments by relying on conceptual cues to a greater extent than healthy controls do. Participants with aphasia received an amplified facilitation effect from conceptual event-related cues compared to the control group, whereas naming in the control group showed a tendency to be more facilitated by lexical co-occurrence information, consistent with previous findings regarding neurotypical reliance on lexical information in verb naming (e.g., Willits et al., 2015). These findings suggest that adaptation to alternative and relatively unimpaired information types may facilitate successful word retrieval in adults with aphasia. Further work should continue to assess potential mechanisms that might underlie rational adaptation in aphasic language, as well as the specific psycholinguistic mechanisms by which conceptual information sources may facilitate verb retrieval. This line of research will ultimately help advance neurorehabilitation and speech-language interventions.

## Data Availability Statement

The raw data supporting the conclusions of this article will be made available by the authors, without undue reservation.

## Ethics Statement

The studies involving human participants were reviewed and approved by University of Pittsburgh Institutional Review Board. The patients/participants provided their written informed consent to participate in this study.

## Author Contributions

HD, TW, and MD conceived of the presented idea. HD and TW developed the experimental materials. HD carried out the experiment, supervised by MD and HD performed the computations and WH verified the analytical methods. HD wrote the manuscript with support from TW and MD. All authors discussed the results and contributed to the final manuscript.

## Conflict of Interest

The authors declare that the research was conducted in the absence of any commercial or financial relationships that could be construed as a potential conflict of interest.
