# Supplementary material for: Rational Adaptation in Using Conceptual Versus Lexical Information in Adults With Aphasia
Source: Front Psychol. 2021 Jan 28;12:589930. doi: 10.3389/fpsyg.2021.589930 (PMC7876333; doi:10.3389/fpsyg.2021.589930)
Supplement: Supplementary file 1 [file Data_Sheet_1.docx]

**Supplementary Table 1.** Stimulus list and properties: Conceptual event-related prime condition.

| **verb target** | **conceptual event-related prime** | | | | | | |
| --- | --- | --- | --- | --- | --- | --- | --- |
|  | **noun cue** | **noun type** | **COCA rank** | **COCA freq** | **USF norms FSG** | **USF norms BSG** | **semantic distance** |
| *ANSWER* | knock | patient | >100 | n/a | 0.02 | 0 | 0.734 |
| *BET* | *racetrack* | location | >100 | n/a | n/a | n/a | 0.661 |
| *CHASE* | *fugitive* | patient | >100 | n/a | n/a | n/a | 0.726 |
| *CHEER* | spectator | agent | >100 | n/a | n/a | n/a | 0.928 |
| *CHOP* | *ax* | instrument | >100 | n/a | 0.09 | 0 | 0.667 |
| *CLEAN* | *maid* | agent | >100 | n/a | 0.19 | 0 | 0.653 |
| *CUT* | *butcher* | agent | >100 | n/a | 0.05 | 0 | 0.684 |
| *DELIVER* | mailman | agent | >100 | n/a | 0.03 | 0.011 | 0.863 |
| *DIG* | *sandbox* | location | >100 | n/a | n/a | n/a | 0.851 |
| *DRAW* | marker | instrument | >100 | n/a | 0.03 | 0 | 0.836 |
| *DRINK* | cocktail | patient | >100 | n/a | 0.45 | 0 | 0.550 |
| *DRIVE* | parkway | location | >100 | n/a | 0.07 | 0 | 0.729 |
| *DUST* | *rag* | instrument | >100 | n/a | 0.02 | 0.041 | 0.764 |
| *EXERCISE* | *gym* | location | >100 | n/a | 0.06 | 0.02 | 0.547 |
| *FLY* | *airport* | location | 29 | 165 | 0.06 | 0 | 0.600 |
| *HAMMER* | *carpenter* | agent | >100 | n/a | 0.13 | 0 | 0.707 |
| *HEAT* | radiator | instrument | >100 | n/a | 0.19 | 0.02 | 0.698 |
| *HUNT* | *rifle* | instrument | >100 | n/a | 0.04 | 0 | 0.748 |
| *INVESTIGATE* | *detective* | agent | >100 | n/a | n/a | n/a | 0.637 |
| *LEARN* | university | location | >100 | n/a | 0.04 | 0 | 0.864 |
| *LOCK* | latch | instrument | >100 | n/a | 0.14 | 0 | 0.505 |
| *MEASURE* | *ruler* | instrument | >100 | n/a | 0.41 | 0.169 | 0.712 |
| *OPEN* | jar | patient | >100 | n/a | 0.06 | 0 | 0.799 |
| *PAY* | toll | patient | >100 | n/a | 0.14 | 0 | 0.801 |
| *PERFORM* | *actor* | agent | >100 | n/a | n/a | n/a | 0.770 |
| *PRAY* | preacher | agent | >100 | n/a | 0.01 | 0 | 0.660 |
| *RECYCLE* | aluminum | patient | 38 | 14 | 0.06 | 0.099 | 0.844 |
| *RENT* | lot | patient | >100 | n/a | 0.01 | 0 | 0.771 |
| *RESCUE* | *hero* | agent | >100 | n/a | n/a | n/a | 0.638 |
| *RIDE* | sleigh | patient | >100 | n/a | 0.12 | 0 | 0.612 |
| *ROLL* | marble | patient | >100 | n/a | 0.03 | 0 | 0.893 |
| *SERVE* | *customer* | patient | 27 | 352 | 0.09 | 0.034 | 0.784 |
| *SHAVE* | blade | instrument | 82 | 7 | 0.02 | 0 | 0.736 |
| *SHOP* | plaza | location | >100 | n/a | 0.12 | 0 | 0.854 |
| *SLEEP* | *bedroom* | location | 45 | 118 | 0.18 | 0 | 0.650 |
| *STAB* | pitchfork | instrument | >100 | n/a | 0.02 | 0 | 0.626 |
| *STEAL* | *burglar* | agent | >100 | n/a | 0.09 | 0 | 0.649 |
| *SWEAT* | *sauna* | location | >100 | n/a | n/a | n/a | 0.705 |
| *THROW* | dart | patient | >100 | n/a | 0.08 | 0 | 0.812 |
| *TYPE* | *secretary* | agent | >100 | n/a | 0.11 | 0 | 0.918 |
| *WAIT* | lobby | location | >100 | n/a | 0.11 | 0 | 0.741 |
| *WALK* | cane | instrument | >100 | n/a | 0.02 | 0 | 0.708 |
| *WATCH* | *audience* | agent | >100 | n/a | 0.03 | 0 | 0.776 |
| *WEAR* | outfit | patient | 49 | 213 | 0.03 | 0 | 0.469 |
| *WORK* | *factory* | location | >100 | n/a | 0.11 | 0 | 0.600 |
| *WORSHIP* | synagogue | location | >100 | n/a | 0.02 | 0 | 0.690 |
| *WRAP* | *tinfoil* | instrument | >100 | n/a | 0.15 | 0.017 | 0.632 |
| *WRITE* | pencil | instrument | >100 | n/a | 0.07 | 0.022 | 0.623 |

Notes: Italics denote items used by McRae and colleagues (2005). COCA is the Corpus of Contemporary American English (COCA), a corpus with more than 560 million words (Davies, 2008). USF FSG and BSG norms are forward strength and backward strength measures from cue-to-target or target-to-cue, these are calculated by dividing the number of participants producing a particular response by the number of participants serving in the group norming the word (Nelson et al., 1998). Semantic distance between cue and target words was calculated by snaut (Landauer & Dumais, 1997; Mandera et al., 2017).

**Supplementary Table 2.** Stimulus list and properties: Lexical collocate prime condition.

| **verb target** | **lexical collocate prime** | | | | | |
| --- | --- | --- | --- | --- | --- | --- |
|  | **noun cue** | **COCA rank** | **COCA frequency** | **USF norms FSG** | **USF norms BSG** | **semantic distance** |
| *ANSWER* | research | 11 | 287 | n/a | n/a | 0.873 |
| *BET* | investors | 34 | 73 | n/a | n/a | 0.867 |
| *CHASE* | dreams | 5 | 85 | n/a | n/a | 0.687 |
| *CHEER* | football | 17 | 25 | n/a | n/a | 0.711 |
| *CHOP* | pieces | 13 | 16 | n/a | n/a | 0.663 |
| *CLEAN* | air | 7 | 277 | n/a | n/a | 0.757 |
| *CUT* | pieces | 3 | 2336 | n/a | n/a | 0.606 |
| *DELIVER* | speech | 4 | 372 | n/a | n/a | 0.834 |
| *DIG* | roots | 25 | 64 | n/a | n/a | 0.772 |
| *DRAW* | line | 1 | 2978 | n/a | n/a | 0.678 |
| *DRINK* | drugs | 25 | 180 | n/a | n/a | 0.707 |
| *DRIVE* | prices | 8 | 454 | n/a | n/a | 0.916 |
| *DUST* | top | 9 | 19 | n/a | n/a | 0.796 |
| *EXERCISE* | diet | 2 | 626 | n/a | n/a | 0.609 |
| *FLY* | sparks | 16 | 238 | n/a | n/a | 0.665 |
| *HAMMER* | details | 3 | 35 | n/a | n/a | 0.907 |
| *HEAT* | cheeks | 94 | 19 | n/a | n/a | 0.813 |
| *HUNT* | food | 4 | 139 | n/a | n/a | 0.715 |
| *INVESTIGATE* | research | 3 | 390 | n/a | n/a | 0.748 |
| *LEARN* | mistakes | 15 | 1050 | n/a | n/a | 0.763 |
| *LOCK* | eyes | 3 | 203 | n/a | n/a | 0.772 |
| *MEASURE* | sensors | 36 | 110 | n/a | n/a | 0.735 |
| *OPEN* | markets | 20 | 519 | n/a | n/a | 0.924 |
| *PAY* | attention | 1 | 15511 | n/a | n/a | 0.691 |
| *PERFORM* | tasks | 1 | 1090 | n/a | n/a | 0.701 |
| *PRAY* | peace | 8 | 202 | n/a | n/a | 0.617 |
| *RECYCLE* | water | 2 | 78 | n/a | n/a | 0.854 |
| *RENT* | month | 19 | 81 | n/a | n/a | 0.544 |
| *RESCUE* | fire | 5 | 179 | n/a | n/a | 0.598 |
| *RIDE* | storm | 16 | 165 | n/a | n/a | 0.727 |
| *ROLL* | hall | 33 | 117 | n/a | n/a | 0.811 |
| *SERVE* | purpose | 1 | 1844 | n/a | n/a | 0.605 |
| *SHAVE* | strokes | 7 | 65 | n/a | n/a | 0.798 |
| *SHOP* | antiques | 10 | 77 | n/a | n/a | 0.695 |
| *SLEEP* | night | 1 | 3601 | n/a | n/a | 0.381 |
| *STAB* | back | 1 | 223 | n/a | n/a | 0.630 |
| *STEAL* | election | 4 | 295 | n/a | n/a | 0.944 |
| *SWEAT* | palms | 5 | 84 | n/a | n/a | 0.760 |
| *THROW* | money | 4 | 838 | n/a | n/a | 0.670 |
| *TYPE* | commands | 10 | 34 | n/a | n/a | 0.938 |
| *WAIT* | second | 2 | 5807 | n/a | n/a | 0.430 |
| *WALK* | door | 1 | 2136 | n/a | n/a | 0.612 |
| *WATCH* | television | 7 | 1682 | n/a | n/a | 0.542 |
| *WEAR* | hair | 13 | 557 | n/a | n/a | 0.647 |
| *WORK* | system | 9 | 1916 | n/a | n/a | 0.827 |
| *WORSHIP* | freedom | 6 | 79 | n/a | n/a | 0.787 |
| *WRAP* | boots | 6 | 222 | n/a | n/a | 0.867 |
| *WRITE* | name | 18 | 904 | n/a | n/a | 0.698 |

Notes: Italics denote items used by McRae and colleagues (2005). COCA is the Corpus of Contemporary American English (COCA), a corpus with more than 560 million words (Davies, 2008). USF FSG and BSG norms are forward strength and backward strength measures from cue-to-target or target-to-cue, these are calculated by dividing the number of participants producing a particular response by the number of participants serving in the group norming the word (Nelson et al., 1998). Semantic distance between cue and target words was calculated by snaut (Landauer & Dumais, 1997; Mandera et al., 2017).

**Supplementary Table 3.** Stimulus list and properties: Unrelated baseline prime condition.

| **verb target** | **unrelated baseline prime** | | | | | |
| --- | --- | --- | --- | --- | --- | --- |
|  | **noun cue** | **COCA rank** | **COCA frequency** | **USF norms FSG** | **USF norms BSG** | **semantic distance** |
| *ANSWER* | food | >100 | n/a | n/a | n/a | 0.869 |
| *BET* | air | >100 | n/a | n/a | n/a | 0.823 |
| *CHASE* | strokes | >100 | n/a | n/a | n/a | 0.832 |
| *CHEER* | antiques | >100 | n/a | n/a | n/a | 0.972 |
| *CHOP* | attention | >100 | n/a | n/a | n/a | 0.956 |
| *CLEAN* | sparks | >100 | n/a | n/a | n/a | 0.818 |
| *CUT* | freedom | >100 | n/a | n/a | n/a | 0.865 |
| *DELIVER* | diet | >100 | n/a | n/a | n/a | 0.966 |
| *DIG* | speech | >100 | n/a | n/a | n/a | 0.962 |
| *DRAW* | storm | >100 | n/a | n/a | n/a | 0.907 |
| *DRINK* | back | >100 | n/a | n/a | n/a | 0.596 |
| *DRIVE* | fire | >100 | n/a | n/a | n/a | 0.811 |
| *DUST* | money | >100 | n/a | n/a | n/a | 0.805 |
| *EXERCISE* | research | >100 | n/a | n/a | n/a | 0.911 |
| *FLY* | details | >100 | n/a | n/a | n/a | 0.944 |
| *HAMMER* | second | >100 | n/a | n/a | n/a | 0.873 |
| *HEAT* | purpose | >100 | n/a | n/a | n/a | 0.896 |
| *HUNT* | hall | >100 | n/a | n/a | n/a | 0.900 |
| *INVESTIGATE* | night | >100 | n/a | n/a | n/a | 0.816 |
| *LEARN* | peace | >100 | n/a | n/a | n/a | 0.841 |
| *LOCK* | month | >100 | n/a | n/a | n/a | 0.877 |
| *MEASURE* | markets | >100 | n/a | n/a | n/a | 0.983 |
| *OPEN* | pieces | >100 | n/a | n/a | n/a | 0.856 |
| *PAY* | eyes | >100 | n/a | n/a | n/a | 0.853 |
| *PERFORM* | roots | >100 | n/a | n/a | n/a | 0.932 |
| *PRAY* | prices | >100 | n/a | n/a | n/a | 0.842 |
| *RECYCLE* | line | >100 | n/a | n/a | n/a | 1.039 |
| *RENT* | boots | >100 | n/a | n/a | n/a | 0.865 |
| *RESCUE* | palms | >100 | n/a | n/a | n/a | 0.924 |
| *RIDE* | name | >100 | n/a | n/a | n/a | 0.774 |
| *ROLL* | door | >100 | n/a | n/a | n/a | 0.841 |
| *SERVE* | sensors | >100 | n/a | n/a | n/a | 1.013 |
| *SHAVE* | drugs | >100 | n/a | n/a | n/a | 0.922 |
| *SHOP* | football | >100 | n/a | n/a | n/a | 0.898 |
| *SLEEP* | pieces | >100 | n/a | n/a | n/a | 0.870 |
| *STAB* | hair | >100 | n/a | n/a | n/a | 0.899 |
| *STEAL* | system | >100 | n/a | n/a | n/a | 0.856 |
| *SWEAT* | tasks | >100 | n/a | n/a | n/a | 0.915 |
| *THROW* | top | >100 | n/a | n/a | n/a | 0.817 |
| *TYPE* | investors | >100 | n/a | n/a | n/a | 1.011 |
| *WAIT* | commands | >100 | n/a | n/a | n/a | 0.783 |
| *WALK* | research | >100 | n/a | n/a | n/a | 0.977 |
| *WATCH* | mistakes | >100 | n/a | n/a | n/a | 0.789 |
| *WEAR* | cheeks | >100 | n/a | n/a | n/a | 0.829 |
| *WORK* | television | >100 | n/a | n/a | n/a | 0.773 |
| *WORSHIP* | election | >100 | n/a | n/a | n/a | 0.930 |
| *WRAP* | dreams | >100 | n/a | n/a | n/a | 0.834 |
| *WRITE* | water | >100 | n/a | n/a | n/a | 0.877 |

Notes: Italics denote items used by McRae and colleagues (2005). COCA is the Corpus of Contemporary American English (COCA), a corpus with more than 560 million words (Davies, 2008). USF FSG and BSG norms are forward strength and backward strength measures from cue-to-target or target-to-cue, these are calculated by dividing the number of participants producing a particular response by the number of participants serving in the group norming the word (Nelson et al., 1998). Semantic distance between cue and target words was calculated by snaut (Landauer & Dumais, 1997; Mandera et al., 2017).

Supplementary Figure 1. Posterior predictive check for Model 1: Distribution of observed and replicated Ys.


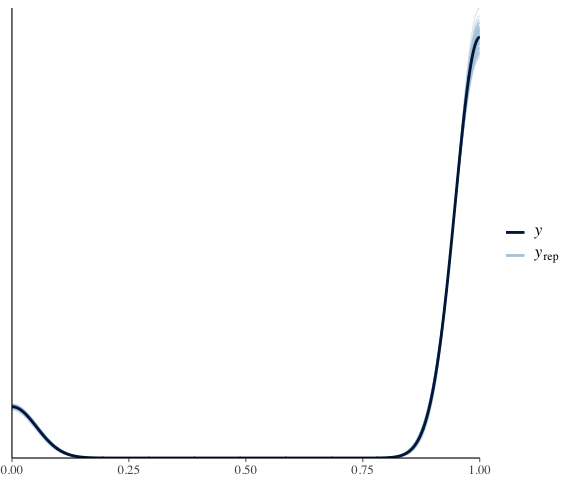


Supplementary Figure 2. Posterior predictive check for Model 2: Distribution of observed and replicated Ys.


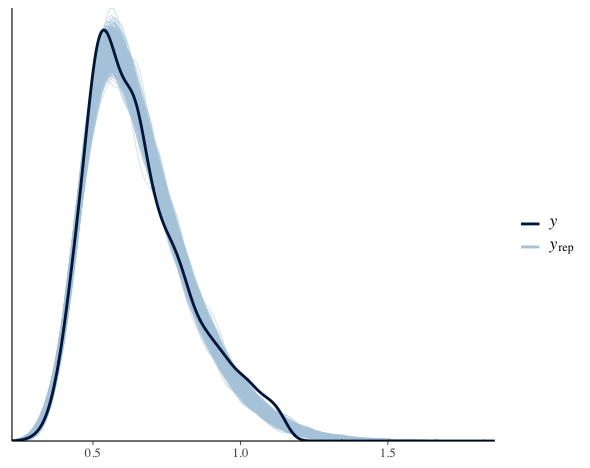


Notes: *Y* reflects an expected bimodal distribution between participants with aphasia and controls. The upper tail reflects the fact that the model does well fitting performance except for participants with aphasia at the upper tail of the response time distribution. Further work is required to understand the extent to which this poor fit is due to a lack of data observations of response times at this tail of the distribution.

Supplementary Figure 3. Posterior predictive check for Model 3: Distribution of observed and replicated Ys.


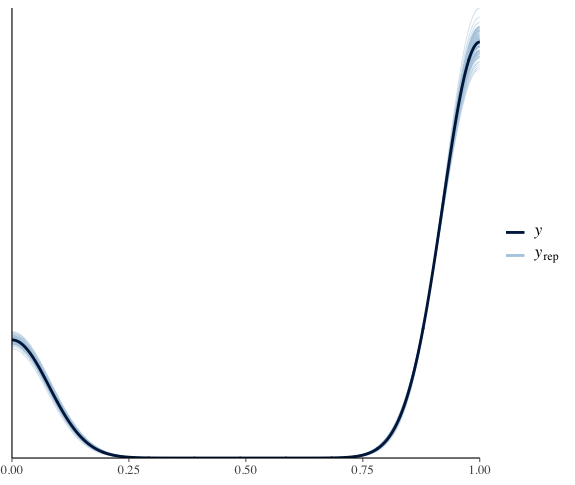


**Supplementary Figure 4.** Posterior predictive check for Model 4: Distribution of observed and replicated Ys.


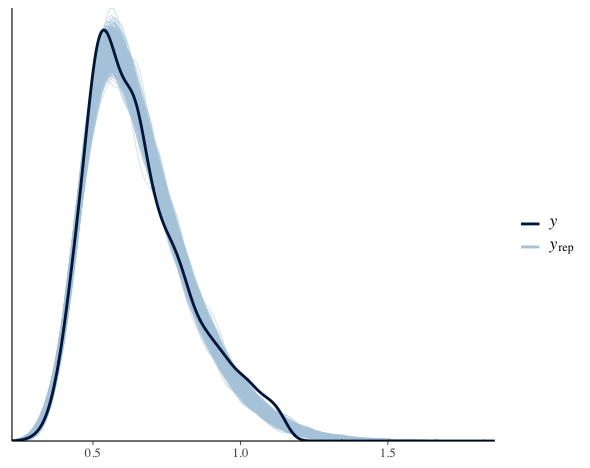


Notes: *Y* reflects the fact that the model does well fitting participant performance except for participants with aphasia at the upper tail of the response time distribution. Further work is required to understand the extent to which this poor fit is due to a lack of data observations of response times at this tail of the distribution.
